# Mr. Wilson, on Mrs. Haslewood's Case

**Published:** 1802-09-01

**Authors:** John Wilson

**Affiliations:** Gillingham


### *To the Editors of the Medical and Phyfical Journal.*

Gentlemen,

When I had the pleafure to tranfmit to you the Report of Mrs. Haflewood's cafe, I could not fufpect that any part of my Paper would excite difpleafure in the mind of any party concerned; but Mr. Congreve deemed himfelf injured by a paffage that he thought admitted of an invidious interpretation; it is as follows: "About a week after, on the annual change of parifh officers officers taking place, I was nominated to the fuperintendance of the poor, and felt myfelf happy in the opportunity afforded me of inveftigating this cafe by a fcrutinizing inquiry."

This was fuppofed to imply a deficiency of talent or attention in Mr. C. I am, Gentlemen, too jealous of my profeffional honour to permit any man to trifle with it; and have, I hope, too juft a fenfe of what I owe my brethren to make a wilful and unprovoked attack on the character of a medical man. I had not the moft remote caufe to cenfure Mr. C.; and all I meant by the paffage alluded to was, that as the cafe was extremely obfcure, I was glad to have it under my own immediate controul. Another paffage too has been confidered exceptionable, and is fuppofed to imply that Mr. Congreve, as well as the Gentlemen of the Surrey Difpenfary, and of Guy's Hofpital, are all culpable for not performing fome operation to relieve the patient; it runs thus: "What the event of an earlier operation might have been we can only furmife, &c. &c." But let it be attended to, that the cafe was under my own care for fome time before I thought proper to operate, and therefore the paffage is as applicable to myfelf as to others, which confideration reduces the fuppofition to an abfurdity.

Confcious as I am that I never intended a perfonality, I think myfelf peculiarly unfortunate in being fo egregioufly mifunderftood. I affure you, Gentlemen, that if an unhappy *necessity* ever fhould arife to expofe the incapacity or inhumanity of a fellow member of the Faculty, I will do it boldly, without referve or inuendo, without refpect to perfons or authority; but I difclaim every prefumption of improper treatment in the conduct of Mr. C. or any of thofe gentlemen in whofe cafe Mrs. Haflewood was, previous to her coming into Gillingham poor-houfe. Her cafe was fo truly intricate as to juftify any diverfity of opinion, or peculiarity of management.

Difmiffing this unwelcome fubject, I now beg leave to introduce the following letter, which I laft night received from Dr. Vaughan; and as it may throw fome light on Mrs. Haflewood's cafe, I offer it to your attention, requefting you will infert it.

"Dear Sir, *Rochester, August* 10, 1802.

I am pleafed to find that, after examining the body of Mrs. Haflewood, you have thought it a duty incumbent upon you to draw up a ftatement of her whole cafe.

All that fell under the cognizance of my fenfes when I paid her a vifit at your requeft, and all that fhe then related concerning her diforder, was not fufficient ground for me whereon to to found a judgment. The hiftory of her difeafe may be thus began with the account given by her fifter: "Six months ago fhe had a fever, after which fhe did not menftruate, and the whole abdomen became enlarged; but on lifting too heavy a weight, fhe brought on an exceffive flow of menfes for a fortnight; and immediately preceding that time her thigh and leg began to fwell," p. 47. But even in this exordial account there arifes a doubt, whether the ftoppage of the menfes, and the enlargement of the whole abdomen, were connected as caufe and effect? And alfo, whether this enlargement of the abdomen was removed as foon as the menfes had flowed exceffively for a fortnight?

There would, I believe, have been lefs obfcurity in Mrs. Haflewood's cafe, if fhe herfelf had not, all along, confounded the two fwellings of the *abdomen* with each other; and if fhe had not forgotten to mention the fever that had preceded the fwelling of the whole *abdomen*, or the too heavy weight that fhe had lifted before the fwelling of the thigh and leg commenced; for the three fwellings were, it would feem, diftinct, and proceeding from different caufes. The fwelling of the whole *abdomen* was the firft in order, and it was probably *ascites*; the fwelling of the thigh and leg was next in order, and it was *oedema*; the third and laft was that fwelling in the right fide of the *abdomen*, which you found as big as a fift, and rightly imagined to be encyfted. Whether thefe three difeafes were at any time all of them co-exiftent or not, we are not informed; but that they became fo at laft, although in different degrees, is, fince the Diffection, highly probable.

And yet there would have been confiderable obfcurity, let the fymptoms have been ever fo minutely detailed, and the particular relation of them, one to another, have been ever fo impreffively ftated. Thus, as the fwelling of the whole *abdomen* was not obferved by either you or me, we know not what we fhould have thought of it. Could we have referred it to that fuppreffion of the *menses*, which immediately preceded it; or to the fever, which immediately preceded the fuppreffion of the *menses?* I am now difpofed to fufpect that there was fome difeafe of the *peritonæum*, or of the liver or fpleen; that hydatids were formed in the *abdomen*, and that their burfting and difcharging their contents produced a kind of *ascites*. (See Medical Tranf. ii. 497.) This is conjecture I admit; but how often do we meet with patients complaining of fever only, in whom we difcover quickly and clearly the fymptoms or confequences of inflammation. It is not vague conjecture then; nor can it be deemed improbable, when it is reflected that your trocar made an outlet for *serum*, containing fragments of hydatids,—

tids,—"flocules refembling the coagulated lymph often found in the abdomen, &c." "A portion of membrane refembling part of a fac or cyft," p. 46; and that the tumour in the *vagina* (Denman's Midwifery) as well as that of the abdomen, fubfided after your operation.

Again, the fwelling of the thigh and leg came on a fortnight exactly after the commencement of the uterine hæmorrhage. Did this fwelling enfue from a ftrain in lifting the heavy weight? If fo, why did it not appear fooner? If it depended on the hæmorrhage, why did not both thighs fwell, and indeed the whole body?

And again, Mrs. Haflewood had always complained of a pain in her loins; during the fever; during the fubfequent fwelling of the whole *abdomen*; after the lifting of the weight too heavy for her ftrength; during the hæmorrhage; and, in a word, till death put an end to it and her life. This pain was not exaggerated after fhe had lifted the weight; therefore, as the fwelling of the thigh was evidently not owing to any matter that had defcended with the *psoas* mufcle under Poupart's ligament, but to lymph in the reticular membrane; and as the pain of the loins did not feem to be at all connected with this fwelling of the thigh and leg; and as no fwelling had yet appeared in the right fide of the *abdomen*, there was nothing like a reafon for fuppofing the exiftence of a *psoas* abfcefs. There was an *œdema* of the thigh and leg, and blifters had accordingly been applied. What internal medicines had been directed while fhe ftaid in London, fhe knew not; but we may fairly conclude, that the object of them was to increafe abforption, and to reftore ftrength.

To proceed with the hiftory. A fhort time after the thigh and leg began to enlarge, fhe felt a hard body, nearly the fize of a large marble, in the fame fide of the abdomen, p. 45. Here there is evidently a chafm, but I fuppofe not a great or material one, between Mrs. Haflewood's account of this tumour and your account of it. I fhall continue to quote your words, as the Medical and Phyfical Journal may not be at hand. "The tumour of the abdomen, which had been rapidly increafing, was then about the fize of the hand when clenched; it evidently contained a fluid, and was fo circumfcribed in its external form as to leave but little doubt as to its being contained in a fac or cyft. It was fituated above the tendon of the oblique mufcle, ftretching from thence obliquely upwards, and towards the ileum: it was not difcoloured. The thigh, which was bent towards the abdomen, was much enlarged; the leg alfo was fwelled, very painful, and the knee bent. The tumour was not fo exquifitely tender; nor did fhe at any time fuffer fo much much pain there as in the extremity. She complained of pain in the loins; ſhe could not paſs urine except when on her hands and knees, and then in ſmall qnantity, and much diſtreſs. She had not any inteſtinal evacuation, but when an enema was adminiſtered; ſhe had not menſtruated ſince the commencement of her complaint. Her breaſts were abſolutely gone; her pulſe was ſmall and quick: ſhe was much emaciated; had the hippocratic countenance; and without the aid of opiates, her nights were ſleepleſs." Page 44. Now this is not the commoneſt preſentation for a *psoas* abſceſs, becauſe the purulent matter deſcending through the reticular membrane makes a tumour commonly in the thigh by elevating its *fascia*, or near the loins, or at the hip-joint, or in the groin, or ſomewhere about the *rectum*. Mrs. Haſlewood's caſe, however, offers, in this particular, no knot which we cannot eaſily looſen. All know that matter formed near the *psoas* muſcle may inſinuate itſelf between the three *strata* of abdominal muſcles; and I think it is moſt likely to do ſo, when the patient is, as Mrs. Haſlewood had long been, almoſt entirely confined by a previous diſeaſe to a horizontal poſition of the body; and a horizontal poſition, or even preſſure, did not leſſen the tumour; nor did a cough give the perception of a fluid impelled from the loins; nor were the kidney and ureter of the right ſide only affected; all which circumſtances generally concur to characteriſe a *psoas* abſceſs. Beſides, the inflammatory ſymptoms muſt have been unuſually ſlight; and the ſuppurative proceſs does not ſeem to have been even felt by the patient herſelf; for of neither is the leaſt mention made in the hiſtory of her caſe.

In your relation of the morbid appearances, you ſay, "The iſchium from the ſpinous proceſs upwards, the ſacrum, and the lumbar vertebrae, where ſome pus was lodged, were all carious." Surely, *caries* of the adjacent bones is no common effect of the matter of a lumbar abſceſs. I cannot, I declare, help thinking that the pus here collected, was rather the effect of the carious *vertebrae*; and the pain you deſcribe, "as cauſed by preſſure or diſeaſe of the iſchiadic nerve," I ſuſpect would ſhortly, if ſhe had lived, ended in a palſy of both the lower extremities.

Before I quit this ſubject, I muſt obſerve, that you ſpeak of "a condenſation of the cellular membrane forming the parietes of the tumour, &c. p. 47. Do you not believe the cyſt of every abſceſs to be formed by what Mr. Hunter calls adheſive inflammation?

I am now come to conſider, not your opinion that Mrs. Haſlewood had an ovarian dropſy, (enough having been already advanced concerning the ſymptoms of her diſeaſe, and alſo concerning cerning their agreement with the morbid changes of ſtructure,) but your animadverſions on my objections to that opinion.

Speaking of me, you ſtate, "I mentioned to the Doctor my opinion of its being an ovarian dropſy, and thought that opinion ſtrengthened by the circumſtance of her having loſt her breaſts, and ceaſed to menſtruate about the beginning of her complaint; but he did not coincide with me in idea that the ovaria were ſtrictly neceſſary to menſtruation, and moreover urged that the left ovarium offered no appearance of diſeaſe." p. 45.

The fact is, I did not aſſent to your opinion, becauſe the ſymptoms of her diſeaſe did not ſeem to me to be ſuch as diſtinguiſh the dropſy of an *ovarium.* And as for the ſubſidence of her breaſts, and the ceſſation of menſtruation, which you adduced in ſupport of your opinion; the former was, I thought, only a part of the general affection of the ſyſtem, for ſhe was miſerably emaciated; and the latter was not noticed, as I could recollect, by any good author as a pathagnomonic ſign of this dropſy. An irregularity in menſtruation does indeed occur in the advanced ſtage of this diſeaſe, but then it is only a ſymptom of cachexy. Mention is made by Morgagni from Gullman, of a virgin, whoſe *menses* were regular, although ſhe had laboured under the dropſy of an *ovarium* for fifteen years; and from Targioni, of a matron who ſuffered this diſeaſe thirty-four years, in whom the *menses* were exceſſive. A perſon may have the dropſy of an *ovarium* for a long time, without the health being much impaired; thus Morgagni relates a caſe, in which after the dropſy was evinced by a tumor in the abdomen, the woman conceived, was happily delivered, and remained long afterwards alert, ſprightly, and robuſt. (*De Sedibus et Causis Morborum, &c. Ep.* 38, *Art.* 64.)

The words "but he did not coincide with me in idea that the ovaria were ſtrictly neceſſary to menſtruation, and moreover urged that the left ovarium offered no appearance of diſeaſe," ſeem to me to convey a meaning ſomewhat ambiguous. I confeſs, I do not underſtand them. I believe the *menses* to be a ſecretion from the arteries of the *uterus* itſelf, i. e. the hypogaſtric; and this belief I publiſhed more than eleven years ago, in the notes to my tranſlation of *Leber's Prælectiones Anatomicæ.* In the ſame notes too, I adopted the opinion of Mr. Hunter, that the *ovaria* and their appendages conſtitute the female ſex, (vol. ii.) and I never dreamt that the *ovaria*, the Fallopian tubes, and the *uterus*, were not in a ſimultaneous action during a fruitful coition. Really then, if you think, that the total deſtruction of the *ovaria* will ſtop menſtruation and induce barrenneſs, I never thought otherwiſe. But this does not not prove, that the *ovaria* have more to do with menftruation than the Fallopian tubes have. If the Fallopian tubes be divided, fo that the *ovum* has no paffage for its defcent to the *uterus*, the *ovaria*, becaufe rendered ufelefs, will fhrink; the inclination for the male will ceafe; nor will even the *uterus* form a *membrana decidua*, though coition be performed ever fo vigoroufly.

It is plain then, why I told you that one of the *ovaria* manifefted no morbid action; one tefticle is enough for generation, and fo is one Fallopian tube. Experience as well as analogy proves this; for Mr. Hunter found that when one *ovarium* was taken away, the conftitution made the remaining one produce its number of young in a lefs time, (Phil. Tranf. vol. lxxvii.); and another Anatomift has difcovered, that when one Fallopian tube is deftroyed, there is no impediment to conception; nay, *corpora lutea* may be feen in the ufelefs *ovarium*.

I am, &c.

W. VAUGHAN."

The firft point that I beg leave to notice in the Doctor's letter is, his doubt refpecting my meaning when I fpeak of "a condenfation of the cellular membrane forming the parietes of the tumor, &c. &c." By this condenfation I wifhed to be underftood the boundary of the abfcefs on that fide, and to denote that the rectum, vagina, Fallopian tube, ovary, and uterus, were not *immersed* in *matter*, but were protected by cellular membrane. Whether the parietes of an abfcefs be the effect of inflammatory adhefion, or of condenfation, owing to the abforption of the fluid contained in the cellular membrane, produced by preffure, may admit of fome controverfy, although the modern Oracle of Delphos has pronounced the former as univerfal.

I think myfelf indifpenfably called upon to ftate my reafons in fupport of the opinion I have long entertained, that the ovaria are the organs of menftruation, becaufe a part of the Doctor's letter is founded on a belief that my conjecture is erroneous.

Before I proceed however, I beg to be underftood, that the principal circumftances in Mrs. H's cafe, on which I grounded my idea that it was an ovarian dropfy, were, the form and fituation of the tumor. Other concomitant fymptoms I thought corroborated my conjecture. Amongft them I numbered the ceffation of the menfes and the flaccidity (or rather *annihilation*) of the breafts. The ovarium of the left fide, I prefumed, had ceafed its functions by fympathy with the difeafed one of the right, never doubting the *possibility* of fuch affociation. But to revert to the fubject in queftion.

The

The Reafons for my fuppofition, and to which I wifh to call the attention of Phyfiologifts, are as follow. When a fow or bitch is fpayed, fhe never fecretes that difcharge which allures the male and excites to copulation. May not this fecretion be the effect of a vafcular orgafm, bearing fome analogy to the menfes; for if it was vaginal, or uterine, the extirpation of the ovaria could not prevent its periodical recurrence. Does not a venereal orgafm take place in birds at the vernal period? and in moft other oviparous animals at feafons peculiar to the refpective organizations? I do prefume, (but with a refpectful deference to my fuperiors in information) that what is termed the uterus in the oviparous tribes is not properly fuch; it performs fcarcely any of the functions common to the viviparous uterus; it is merely a receptacle for the ovum until the parts of the embryon are formed; the ovum is then caft off, and the augmentation in bulk, with the perfection of organization, are left to chance or accident. But although they have not diftinct and proper uteri, in the ftrict acceptation of the term, they have ovaria, and thefe I prefume are the feat of their venereal orgafm. It is, I believe, univerfally acknowledged, that a female who never menftruates is always fterile, and that the period of menftruating power is correfpondent with the generating power. From about the age of fourteen to forty-five years, conftitutes the general limit of thefe abilities in this climate.

Let us now combine the following facts:

Firft, Viviparous and oviparous animals cannot generate their fpecies without the ovaria.

Second, Many viviparous animals are fubject to periodical venereal orgafm, which is prevented by extirpation of the ovaria.

Third, The generating and menftruating powers are coeval and coexiftent in the human fubject.

If then, in the firft inftance, the ovaria are neceffary to propagation, and in the fecond to venereal orgafm, do we not, by aid of the third, approximate to an analogy between the menftrual fecretion of the human, and the venereal orgafm of the brute animal? If this analogy is fuppofed to be eftablifhed, I prefume we may conclude, that as in the firft inftance, generation cannot take place without the ovaria; in the fecond, they are requifite to venereal orgafm, or that fecretion which feems to correfpond with the propagating propenfity or power; and in the laft, thofe powers are correlative; fo the menftrual fecretion of the human fubject, having an equal correfpondence with the power of generation, is alfo dependent on the ovaria. Quod erat demonftrandum. Do not thefe effects feem to arife from an identity of caufe, that is, a peculiar indifpenfable action of the ovaria? May not the ovaria and their appendages be in fuch a ftate as to be ineffectual to the purpofes of menftruation and and generation? Thus, then, I would account for the concomitance of thefe powers.

A female may be barren from a variety of caufes, and unfortunately it can feldom be afcertained whether the defect caufing fterility originates in herfelf or the male; but I am much inclined to believe, that it is moft frequently imputable to the former. The dropfy of the ovarium, Dr. Denman fays, occurs more commonly at the time of the ceffation of the menfes than at any other period. It has immediately fucceeded fuppreffion of that fecretion. I may add too, that at the final ceffation the ovaria become contracted and rather indurated. All of which circumftances have a tendency to fortify our propofition.

In the cafes adduced by Dr. Vaughan, to prove the fallacy of my opinion, *one* ovarium only was difeafed; and his other obfervations fhew the poffibility of a folitary organ being in fome cafes efficient to the performance of functions that both were intended to effect. I ought, indeed, to thank the Doctor for the additional teftimony that he has been pleafed to afford me, that when the ovaria are taken away, menftruation ceafes and barrenefs enfues. I could not wifh for fairer evidence to eftablifh my conjecture, particularly as it comes from a gentleman not difpofed to favor it with his fanction. Surely this acknowledgment muft militate, in a formidable degree, againft his conclufion, that the menftrual difcharge comes from the arteries of the uterus itfelf! Mr. Pott, who had no particular hypothefis to fupport, and whofe reputation ftood too high to require the artificial aid of candle-light cafes, records the hiftory of a young woman who menftruated regularly before fhe had both the ovaria extirpated in confequence of ovarian hernia, but fubfequent to the time of the operation fhe ceafed to menftruate in any degree. This is a cafe in point, which I judge juftifies my ideas on the fubject under confideration, for had an experiment been inftituted for the fole purpofe of afcertaining the matter in queftion, it could not have been more happily devifed, nor could it have been more decifively favourable to my conjecture.

Thefe facts, Gentlemen, imprefs on my mind a belief that the ovaria are the organs of menftruation, as well as of the fabrication of the ovum. I muft, however, beg my readers to confider any imperfection in the above doctrine as in fome meafure imputable to the natural intricacy of the fubject, and hope that if it is thought worthy of notice, it will be confidered with impartiality, and combated with candor. If I am wrong, I will gratefully acknowledge my errors when conviction is offered me, and hold that gentleman my beft friend, who is moft fuccefsful in detecting my erroneous pofitions. Be the event what it it may, I ſhall be very happy if what I have noted leads to a full, juſt, and diſpaſſionate diſcuſſion of the queſtion, as the reſult may afford us more accurate ideas of menſtruation, and thereby enable us to treat with greater certainty of ſucceſs, at leaſt one of the manifold afflictions incident to the ſolacing partner of mankind.

It would be treſpaſſing too much on your time to lengthen this Communication by a detail of the manner in which I conceive the ovaria are effective to the menſtrual evacuation; I ſhall therefore defer it till a future opportunity, when I may, perhaps, be able to tranſmit to you an account of an examination, poſt mortem, of another lumbar abſceſs that is now under my care, and which is rapidly advancing the deſtruction of my patient.

I am, &c.

